# Variation in the microbial community contributes to the improvement of the main active compounds of *Magnolia officinalis* Rehd. et Wils in the process of sweating

**DOI:** 10.1186/s13020-019-0267-4

**Published:** 2019-10-22

**Authors:** Qinahua Wu, Dan Wei, Linlin Dong, Yuping Liu, Chaoxiang Ren, Qianqian Liu, Cuiping Chen, Jiang Chen, Jin Pei

**Affiliations:** 1 State Key Laboratory Breeding Base of Systematic Research, Development and Utilization of Chinese Medicine Resources, Chengdu, 611137 Sichuan China; 2 Pharmacy College, Chengdu University of Traditional Chinese Medicine, Chengdu, 611137 Sichuan China; 3 Institute of Chinese Materia Medica, China Academy of Chinese Medical Sciences, Beijing, 100700 China

**Keywords:** *Magnolia officinalis* Rehd. et Wils, Sweating, Microbial communities, High-throughput sequencing, High-performance liquid chromatography, UPLC-Q-Extractive Orbitrap mass spectrometry

## Abstract

**Background:**

*Magnolia officinalis* Rehd. et Wils, commonly called Houpo, has been used for thousands of years in China as a traditional herbal medicine. The primary processing of Houpo requires sweating treatment, which is a special drying process and is considered to be an essential embodiment of high quality and genuine medicinal materials. The sweating of Houpo leads to peculiar changes in the microbial community structure and the content of main active substances (magnolol, honokiol, syringin and magnoflorine). Variation in the microbial community was considered the cause of the change in content of active substances of Houpo, although the microbial taxa responsible for the improvement of content remain unidentified.

**Methods:**

In this study, we used MiSeq high-throughput sequencing methods for partial bacterial 16S rRNA and 18S rRNA gene sequences to compare the bacterial and fungal community structures at different timepoints in the process of sweating. The content of the main active substances (magnolol, honokiol, syringin and magnoflorine) were determined by high-performance liquid chromatography analysis to evaluate the effects of sweating. UPLC-Q-Extractive Orbitrap mass spectrometry (UPLC-QE Orbitrap MS) was used to detection of differential metabolites of unsweated Houpo before and after co-culture with core bacterial solutions.

**Results:**

In this study, the total contents of magnolol (MG) and honokiol (HK) were significantly increased at 4 dp (dp for day PM sample), up to 3.75%, and the contents of syringin (SG) and magnoflorine (MF) were as high as 0.12% and 0.06%, respectively. Bacterial abundance and diversity were higher in the early stage (0 day–2 da; da for day AM sample) than in the later stage (4–5 dp), while fungal abundance was more obvious in the later stage than in the early stage. Positive correlation coefficients revealed that the relative abundance of *Enterobacter* (P < 0.05), *Klebsiella* (P < 0.05), *Weissella* (P < 0.05), *Bacillus* (P < 0.05) and *Candida* (P < 0.05) would be conducive to improving the quality of Houpo. Negative correlation coefficients revealed that the relative abundance of *Actinomycetospora*, *Singulisphaera*, *Mucilaginibacter*, *Deinococcus*, *Gemmatirosa*, *Methylobacterium*, *Sphingomonas*, *Hymenobacter*, *Halomonas* and *Capnobotryella* could be a potential antagonist for the decrease in the quality of Houpo. After co-culture of single core strain and unsweated Houpo, there was no significant difference in the four main active components, but there were other metabolites with significant difference.

**Conclusions:**

Our findings reveal that sweating increased the content of the main active compounds, promoted the relative abundance of potentially beneficial microbes, decreased the abundance of potentially harmful microbes, the core functional genera group together, forming a core microbiome, these genera are dominant across the different stages of the sweating process and contribute to the quality development of the characteristics of Houpo. Meanwhile, this study presented a clear scope for potential beneficial microbes that improve the quality of Houpo.

## Background

The primary processing of Traditional Chinese Medicinal Materials (TCMM) refers to the preliminary treatment and drying of medicinal materials from medicinal plants to form commercial medicinal materials. It is an indispensable and important part of the production process and quality formation of CMM [1]. Long-term production practice and experience have formed a unique and enriched processing method and technical system [2]. The traditional processing of Chinese medicinal materials includes cleaning, slicing, steaming, boiling, rubbing, sweating, and drying, and the choice of different processing methods directly affects the quality of CMM [3]. Among the traditional processing techniques, sweating is a unique treatment method adopted to dry materials and is an important factor in the quality of some medicinal materials, such as root, rhizome, cortex, and sclerotinia [4].

In the sweating process, materials are piled up and the heat is increased so that the internal moisture of the medicinal materials diffuses outwards, thus the term sweating, and the process is finished when the fresh medicinal materials are dehydrated to a certain degree. On the one hand, in the process of sweating, long-term stacking causes internal heat production, resulting in biomass and energy exchange, and the internal water of the medicinal materials is redistributed so that the drying speed is accelerated [4]. On the other hand, changes in the temperature and humidity inside the medicinal materials inevitably leads to changes in the microbial community structure, the activity of enzymes, the regulation and promotion of microbial communities in biological tissues, and the initiation or acceleration of biological and chemical conversion processes of primary/secondary metabolites, and these changes directly influence the formation and the quality of medicinal materials [5]. There are a large number of nonpathogenic microorganisms associated with plants. These microorganisms have coexisted with plants for a long time, which has had a great influence on the formation and content of plant medicinal components [6]. A study in the early 1990s showed that sennoside could be turned into an effective agent for treating diarrhea caused by intestinal bacteria [7]. By optimizing the liquid fermentation conditions for the production of xylanase by *Aspergillus niger* B03, Dobrev increased the xylanase activity to 996.30 U/mL, and its content was increased to 33% of the total proteins [8]. Other studies have also shown that intestinal bacteria convert many of the glycosides, flavonoids, and coumarins contained in TCM into therapeutically useful compounds [9].

Modern research has shown that sweating has an important effect on the character, chemical composition and drug effect of medicinal materials [10, 11]. Meanwhile, the microbial community structure in medicinal materials also changes greatly in the process of sweating. However, the relationship between the quality of TCMM and the change in microbial community structure in the process of sweating has not attracted the attention of scholars. Few studies have reported how the sweating process affects the microbial community in the manner of initiating or accelerating the biological and chemical transformation of primary/secondary metabolites and, as a result, influences the quality of medicinal materials. In this study, we proposed three hypotheses: (1) sweating will impact the quality of medicinal materials; (2) sweating will impact the microbial community composition and diversity; and (3) microbial community is closely related to the quality of medicinal materials and is one of the key factors leading to the change in the content of medicinal materials. Among them, *Magnolia officinalis* Rehd. et Wils (*M. officinalis*) bark can be used as a good model to study the relationship between microorganisms and the quality of TCM.

*Magnolia officinalis* has long been used as a traditional Chinese and Japanese medicine for the treatment of gastrointestinal disorders, anxiety, and bronchial asthma [12, 13], and the stem bark of this plant is known as Houpo. At present, many prescriptions containing Houpo are still in use in modern clinical practice, and *M. officinalis* bark extract is currently listed as a dietary supplement and a major component of cosmetics in many countries [14–16]. In China, *M. officinalis* is usually processed after collection. According to the Chinese Pharmacopoeia (version 2015) (CP 2015) [17], the primary processing of Houpo enforces sweating treatment, called “Fahan”, which is a special drying process and is considered to be an essential embodiment of high quality and genuine medicinal materials. The quality and clinical effect of Houpo are closely related to this process.

In the current Pharmacopoeia of various countries, the evaluation of the quality of commercial Houpo is effectively based on a quantitative determination of the levels of magnolol (MG) and honokiol (HK) in the bark [18, 19]. The CP 2015 [17] and European pharmacopoeia [20] required that MG and HK consisted of a minimum of 2.0% of the total dried herbs. MG and HK are considered the main active constituents and the two principal phenolic compounds in the bark [21, 22] and have been reported to have pharmacological activities with neuroprotective [23], antimicrobial [24, 25], antioxidant [26, 27], antiarrhythmic [28, 29], and anxiolytic effects [30, 31]. In addition to these well-known lignans, alkaloids (magnoflorine, MF), glycosides (syringin, SG), and volatile oils are other interesting secondary metabolites produced by this species [32, 33] are also biologically active [34]. Previous studies have shown that the bioactive content of Houpo was significantly increased after sweating [35], which could enhance gastrointestinal motility and reduce the production of harmful metabolites, allowing Houpo to exhibit its synergistic and detoxifying effects [36].

Previous studies on Houpo mainly focused on the chemical composition [37], pharmacology [38], toxicology [39] and pharmacodynamics [40] of the medicinal materials. However, the relationship between the quality of Houpo and the change of microbial community structure in the process of sweating is not clear. Few studies have reported the effects of microbial community changes on the quality of Houpo. Therefore, it is necessary to further study the relationship between the microbial community and the quality of Houpo in the process of sweating; in other words, determine the response of the microbial community to medicinal quality.

This research adopted high-throughput methods for sequencing partial bacterial 16S rRNA and 18S rRNA gene sequences to compare bacterial and fungal community structures at different timepoints in the process of sweating, and the four main active substances (MG, HK, SG and MF) were determined by HPLC. UPLC-QE Orbitrap MS was used to detection of differential metabolites of unsweated Houpo before and after co-culture with core bacterial solutions. This study aims to provide the following: (i) the identification of the core population of the microbial community in the process of *M. officinalis* sweating, (ii) a better understanding of the relationship between microbial community activity and the quality of Houpo, and (iii) the primary processing of TCMM represented by *M. officinalis* and the role placed by microorganisms in the formation of the quality of medicinal materials.

## Materials and methods

### Sample collection

*Magnolia officinalis* is the second-most important plant species protected in China. Five 12-year-old *M. officinalis* trees with similar physical conditions were collected from a mountain forest located in Xieyuan Town (30.37°N, 103.18°E, 1260 m elevation), Dayi County, Chengdu City, Sichuan Province, China. For each tree, 2 m of stem bark was peeled from the open side of the adret 80 cm above the ground and then used for sweating. Samples were taken twice a day (10 a.m. for the da samples, 4 p.m. for the dp samples, and 0 day for the fresh bark sample) for each biological replicate at different timepoints during sweating treatment. Houpo was piled up sweating at night, ventilated during the day, and repeatedly sweated for 5 day until the end of sweating. Five biological replicates with different sweating times were pooled, ground and divided into two parts. One part was pulverized with liquid nitrogen and stored at − 80 °C for microbial analyses. The other part was dried and used for HPCL content determination. Meanwhile, before the sample sweated, a section of fresh bark was reserved for a − 80 °C to verify the function of the selected core species.

### Analysis of the content of the main active compounds in the process of Houpo sweating

The standard substances, namely, magnolol (MG), honokiol (HK), magnoflorine (MF), and syringin (SG), were purchased from Chengdu ALFA Biotech Company (Sichuan, China). The standard solutions were dissolved in methanol (Fisher, NJ, USA) for further experiments. Five samples of *M. officinalis* from each timepoint were pooled and considered one sample. MG and HK extracts prepared according to the description of the Chinese Pharmacopoeia (version 2015) [17]. SG and MF extracts were prepared according to the description of Gao et al. [41]. The content of four compounds were analyzed with an Ultimate 3000 system (Thermo, USA) using an Agilent Technologies C18 (4.6 mm × 250 mm, 5 µm) column, and the column temperature was maintained at 30 °C. The MG and HK contents were determined with a mixture of methanol/water (78:22) as the mobile phase, and the flow rate and wavelength were 1.0 ml min^−1^ and 294 nm, respectively. A volume of 5 μL was injected for each sample. SG and MF contents were determined with a mixture of acetonitrile/0.2% phosphoric acid (11:89) as the mobile phase, and the flow rate and wavelength were 1.0 ml min^−1^ and 268 nm, respectively. A volume of 5 μL was injected for each sample.

### DNA extraction and PCR amplification

Microbial DNA was extracted from *M. officinalis* cortex samples using the E.Z.N.A.^®^ Soil DNA Kit (Omega Bio-tek, Norcross, GA, U.S.) according to the manufacturer’s protocols. Total DNA concentration and purity were determined by a NanoDrop 2000 UV–vis spectrophotometer (Thermo Scientific, Wilmington, USA). DNA quality was checked by 1% agarose gel electrophoresis as previously reported. The V3–V4 hypervariable regions of the bacterial 16S rRNA gene were amplified by PCR (95 °C for 3 min, followed by 29 cycles at 95 °C for 30 s, 55 °C for 30 s, and 72 °C for 45 s, and a final extension at 72 °C for 10 min) using barcoded primers 338F-806R [42]. The ITS regions of the fungal 18S rRNA genes were amplified by PCR (95 °C for 3 min, followed by 36 cycles at 95 °C for 30 s, 55 °C for 30 s, and 72 °C for 45 s, and a final extension at 72 °C for 10 min) using barcoded primers ITS1F-ITS2R [43]. The PCR system (Gene Amp 9700, ABI, USA) reactions were performed in triplicate in a 20 μL mixture containing 4 μL of 5 × FastPfu Buffer, 2 μL of 2.5 mM dNTPs, 0.8 μL of each primer (5 μM), 0.4 μL of FastPfu Polymerase, and 10 ng of template DNA.

Amplicons were extracted from 2% agarose gels, further purified using the AxyPrep DNA Gel Extraction Kit (Axygen Biosciences, Union City, CA, USA) and quantified using QuantiFluor™-ST (Promega, USA) according to the manufacturer’s protocol.

### Illumina MiSeq sequencing

Purified amplicons were pooled in equimolar ratios and paired-end sequenced (2 × 300) on an Illumina MiSeq platform (Illumina, San Diego, USA) according to the standard protocols by Majorbio Bio-Pharm Technology Co. Ltd. (Shanghai, China). The raw reads were deposited in the NCBI Sequence Read Archive (SRA) database (Accession Numbers: SRP, PRJNA534027 and PRJAN492971).

### Processing of sequencing data

Raw fastq files were demultiplexed, quality-filtered by Trimmomatic and merged by FLASH with the following criteria: (i) The reads were truncated at any site receiving an average quality score < 20 over a 50 bp sliding window. (ii) Primers were exactly matched, allowing 2 nucleotide mismatching, and reads containing ambiguous bases were removed. (iii) Sequences whose overlap was longer than 10 bp were merged according to their overlap sequence.

Operational taxonomic units (OTUs) were clustered with 97% similarity cutoff using UPARSE (version 7.0 http://drive5.com/uparse/) [44], and chimeric sequences were identified and removed using USEARCH [45]. The taxonomy of each 16S rRNA and 18S rRNA gene sequence was analyzed by the RDP Classifier algorithm (version 2.2 http://sourceforge.net/projects/rdp-classifier/) against the Silva (SSU123) database using a confidence threshold of 70% [46].

### Co-culture of different bacterial solutions with unsweated Houpo

Two strains of *Enterobacter*, two strains of *Klebsiella* and one strain of *Bacillus* with significant differences were selected, separated and purified, each 100 μL, and were cultured in 40 mL sterile Luria–Bertani broth [tryptone 10.00 g, sodium chloride 10.00 g, yeast extract 5.00 g, distilled water 1000 mL, high pressure sterilization for 20 min] at 28 °C with shaking (180 rpm) in the dark for 3 day [47], which were labeled as E1, E2, K1, K2 and B, respectively. Meanwhile, the medium without bacteria solution was set as the control group, marked as KB. Each sample had three biological replicates. The fresh bark was cut into small pieces of 2 × 2 cm, then placed in the cultured bacterial solution after high pressure sterilization of 20 min. After shaking for 4 day, the fresh bark was grounded into dry powder with liquid nitrogen.

### Detection of chemical constituents of Houpo treated with bacterial solutions

Briefly, 1.000 g sample was weighed and added with 25 mL methanol for ultrasonic extraction at room temperature for 20 min, then cooled and centrifuged for 12,000 r/min for 5 min, respectively. The supernatant was passed through 0.22-μm microporous membrane and 1 μL aliquots were used for subsequent MS experiments [48].

The UPLC-QE Orbitrap MS (Thermo Fisher Scientific, CA, USA) with a heated electrospray ionization probe was used in this study. Agilent SB-C18 4.6 × 100 μm 1.8 μm column (Agilent, GER)was used to separate the extracts. The solvent system, methanol/water (0.1% acetic acid); gradient program, 20:80 V/V at 0 min, 80:20 V/V at 15 min, 95:5 V/V at 25 min; 95:5 V/V at 30 min, 20:80 V/V at 30.1 min, 20:80 V/V at 35 min. flow rate, 0.2 mL min^−1^; column temperature, 30 °C.

### Statistical analysis

SPSS version 19.0 software (SPSS Inc., Chicago, IL) was used for statistical analyses. Significance was calculated by one-way ANOVA followed by Duncan’s test (P < 0.05), and the values were drawn by Origin software. The microbiological data were analyzed on the free online platform Majorbio I-Sanger Cloud Platform (http://www.i-sanger.com). Sequence analyses were performed using a QIIME platform (http://qiime.org/scripts/assign_taxonomy.html) [49]. A rarefaction analysis including Chao1 estimation and Shannon diversity index were used to calculate the richness and diversity of the microbial communities performed on Mothur 1.30.1 [50, 51], respectively. UniFrac principal coordinate analysis (PCoA) was performed to compare the different timepoints samples on the basis of the weighted UniFrac distance metrics in QIIME and examine dissimilarities in community composition [52, 53]. Meanwhile, linear discriminant analysis (LDA) coupled with effect size measurements (LEfSe, http://huttenhower.sph.harvard.edu/galaxy/root?toolid=lefse_upload) analysis was conducted to search for statistically different biomarkers from 11 timepoints samples [54, 55]. Pearson’s correlation analyses were conducted to investigate the associations of the main active compound contents with the taxonomic diversity and relative abundance of the microbial communities [56–58]. Volcano plot was used to show the changes of chemical composition of Houpo before and after treatment with different bacterial solutions.

## Results

### Analysis of content of the main active compounds in the process of Houpo sweating

**Fig. 1:**
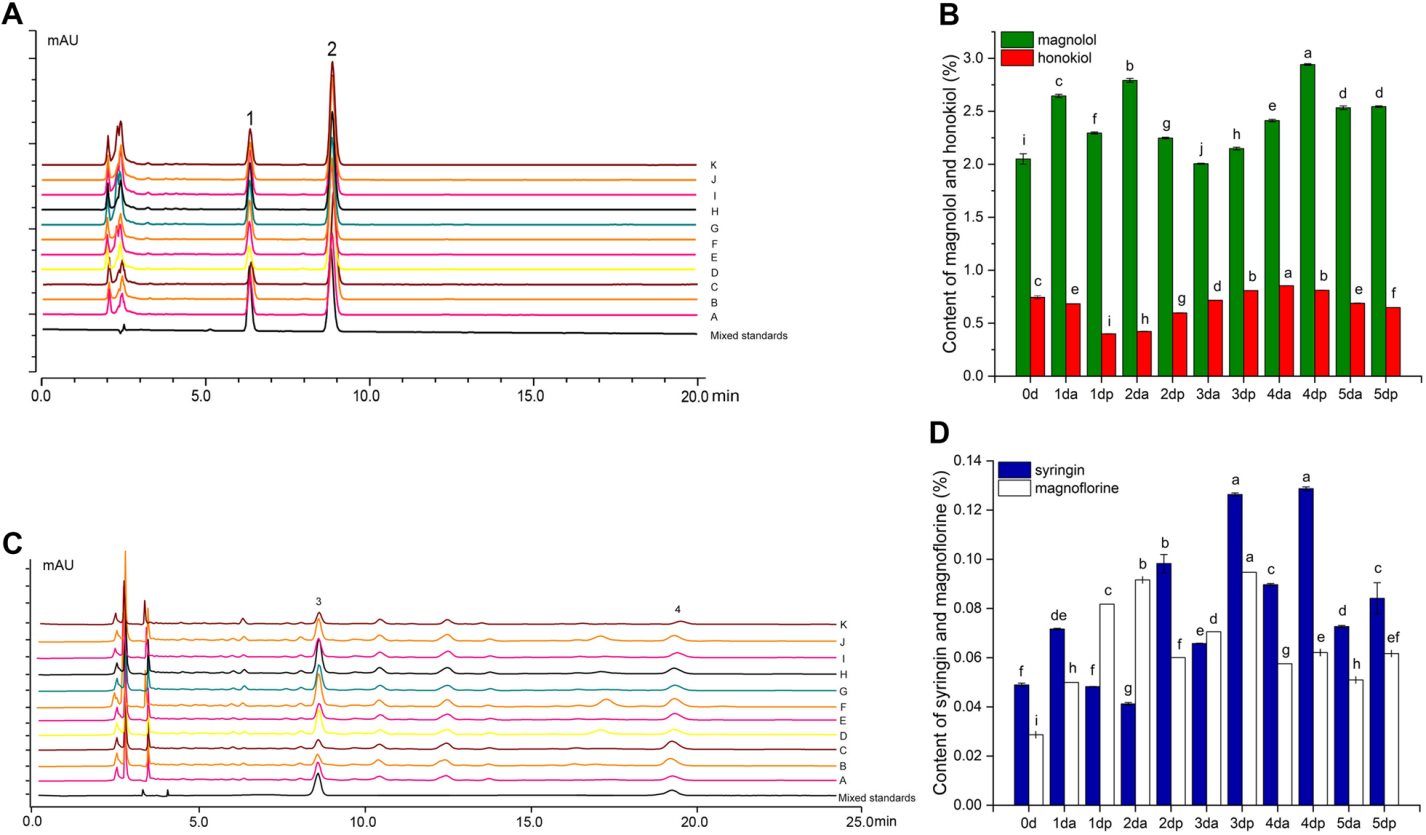
Content of four main compounds in the process of Houpo sweating. **A** HPLC chromatogram of MG and HK of 11 timepoints samples: 1.HK; 2. MG. a–k represent reference substance, 0 day–5 dp samples, respectively. **B** Contents of MG and HK in the process of sweating. **C** HPLC chromatogram of MG and HK of 11 timepoints samples: 3.SG; 4. MF. a–k represent reference substance, 0 d–5 dp samples, respectively. **D** Contents of SG and MF in the process of sweating. Data were presented as mean ± SD (n = 3). Different letters indicate significant differences at P < 0.05 (based on Duncan test)

The content of the main active compounds [magnolol (MG), honokiol (HK), syringin (SG) and magnoflorine (MF)] changed significantly in the process of sweating based on the HPLC analysis (Fig. 1, Additional file 1: Tables S1 and S2). The total amount of MG and HK was over 2.0% during the process of sweating, which was in line with the pharmacopoeia standards. The content of four compounds showed a fluctuating increase, especially MG, SG and MF. At 0 dp–3 da (da for day AM sample, dp for day PM sample), the content of MG fluctuated greatly, showing an overall upward trend and was the lowest at 3 da. Subsequently, the content of MG showed a significant upward trend and was significantly higher than those of other samples at 4 dp, reaching as high as 2.94%. At 5 dp, which was the end of sweating, the content of MG reached 2.54%, which was significantly higher than those of the unsweated samples (0 day, 2.70%). The content of HK decreased at first and then increased as a whole, reaching the lowest value at 1 dp and the highest value at 4 da, up to 0.84%, while at 5 dp, the content was significantly lower than those of the unsweated samples (Fig. 1A, B). The contents of syringin (SG) and magnoflorine (MF) were relatively low; the change trend was not consistent and fluctuated at 0 day–3 da, but the overall trend showed an upward trend. At 3 da–5 dp, the contents of SG and MF were consistent and were significantly higher than those of the other samples at 3 dp. Among 3 da–5 dp samples, the content of MF was the highest at 3 dp, while SG was the highest at 4 dp. The contents of SG and MF were as high as 0.129% and 0.094%, respectively (Fig. 1C, D).

### Diversity of the microbial community

To evaluate the effects of sweating on the microbial community of medicinal materials, we analyzed the changes in the microbial community of Houpo at different sweating timepoints. A total of 1,483,789 and 2,285,109 quality-filtered and chimera-checked 16S/18S rRNA gene sequences were obtained with an average length of 437 bp and 248 bp (Additional file 1: Tables S3 and S4) across all samples, respectively. The number of 16S rRNA sequences obtained per sample varied from 35,665 to 59,049, and the number of fungal 18S rRNA sequences per sample varied from 49,501 to 74,151. In total, 1694 bacterial OTUs and 971 fungal OTUs were obtained from the 33 (11 timepoints × 3 biological replicates) DNA samples.

The bacterial and fungal community diversity (Chao and Shannon) index values from different sweating timepoints were compared (Table 1). The Chao estimator showed that the bacterial community abundances of the 0 day and 1 dp samples were significantly higher than those of the 2–5 dp samples. The bacterial community abundances of the 1 da, 2 da, and 2 dp–4 da samples were significantly higher than those of the 4–5 dp; the 5 dp samples were significantly lower than the 4 dp and 5 da samples. The fungal community abundances of the 0 d, 1–2 dp, and 3 dp samples were significantly higher than those of the 1 da, 3 da, and 4 da–5 dp samples, especially those of the 3 dp samples. Additionally, the fungal abundances of the 3 da and 4 da samples were significantly higher than those of 4–5 dp samples. The Shannon indices showed that the bacterial diversities of the 0 day–2 da samples were significantly higher than those of the 2 dp and 4 da–5 dp samples, and the bacterial diversities of the 3 da and 3 dp samples were significantly higher than those of the 4–5 dp samples. At the same time, the fungal communities of the 0 day–3 dp samples were significantly more diverse than the communities of the 4–5 dp samples, and the most diverse community was that of the 2 da sample. Additionally, the fungal diversities of the 4 da and 5 dp samples were significantly higher than those of the 4 dp and 5 da samples.

**Table 1:** Alpha diversity of bacterial and fungal community in the process of Houpo sweating

Time points	Bacterial		Fungus	
	Chao	Shannon	Chao	Shannon
0 day	292.286 ± 28.035a	2.727 ± 0.118a	90.042 ± 9.578abc	1.994 ± 0.068a
1 da	237.425 ± 62.133ab	2.858 ± 0.114a	73.843 ± 1.838bcd	1.626 ± 0.016ab
1 dp	305.794 ± 13.356a	2.692 ± 0.197a	97.010 ± 7.111abc	1.687 ± 0.103a
2 da	234.899 ± 6.799ab	3.113 ± 0.147a	107.758 ± 19.093ab	2.032 ± 0.046a
2 dp	175.513 ± 11.699bcd	2.038 ± 0.078bc	89.005 ± 4.635abc	1.738 ± 0.034a
3 da	198.229 ± 11.788bc	2.578 ± 0.436ab	64.010 ± 10.959cd	1.483 ± 0.095ab
3 dp	206.983 ± 11.054bc	2.585 ± 0.176ab	119.637 ± 14.931a	1.967 ± 0.054a
4 da	205.653 ± 18.242bc	1.979 ± 0.317bc	60.792 ± 14.382cd	1.025 ± 0.243bc
4 dp	144.143 ± 26.484cde	1.946 ± 0.034c	14.833 ± 3.059e	0.183 ± 0.002d
5 da	110.238 ± 6.016de	1.786 ± 0.116c	7.667 ± 0.881e	0.187 ± 0.040d
5 dp	85.667 ± 6.328e	1.719 ± 0.059c	10.333 ± 0.333e	0.457 ± 0.063cd

0 day represents the cortex without sweating. 1 da, 2 da, 3 da, 4 da, 5 da present day AM samples, 1 dp, 2 dp, 3 dp, 4 dp, 5 dp present day PM samples. Data are presented as mean ± SD (n = 3). Different lowercase letters represent significant differences between different timepoints at *P *< 0.05

### Composition of microbial communities

**Fig. 2:**
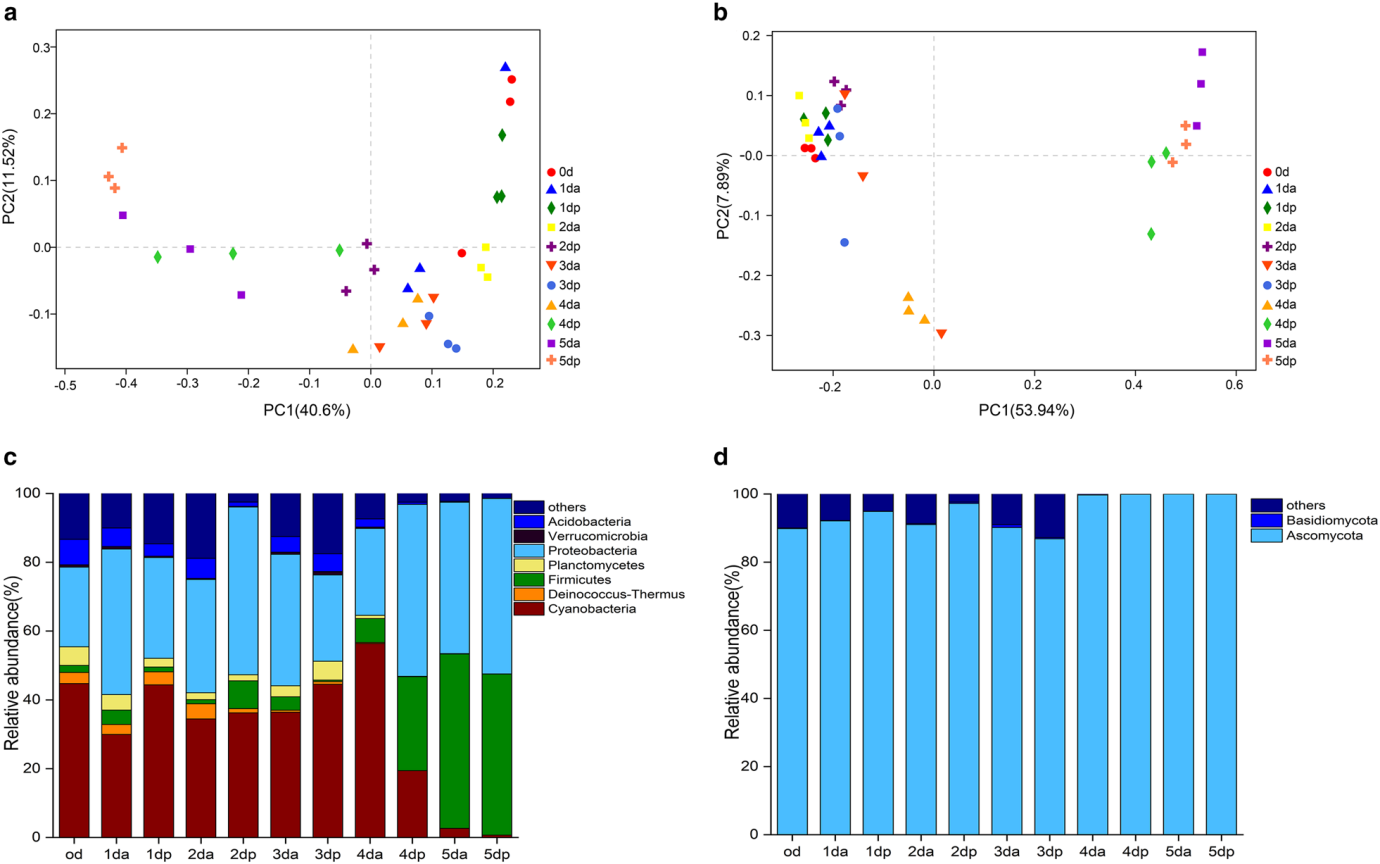
The β diversity of microbial community in different sweating timepoints. Principal coordinates analysis of bacterial (**a**) and fungal (**b**) communities. The values of axes 1 and 2 are the percentages that can be explained by the corresponding axis. **c** Bacterial community at the phylum level in different sweating timepoints, **d** Fungal community at the phylum level in different sweating timepoints

PCoA was used to examine the β-diversity at the genus level. The PCoA clearly grouped the bacterial and fungal communities according to 11 sweating timepoints (Fig. 2a, b). The values of axes 1 and 2 were the percentages explained by the corresponding axis. The first two axes (PC1 and PC2) explained 52.12% and 61.83% of the total bacterial and fungal variation in 33 Houpo samples, respectively. The predominant species in the bacterial communities varied, while the fungal communities were largely consistent in the process of sweating. However, differences in relative abundances were observed (Fig. 2c, d). The bacterial phyla distribution at different sweating timepoints were Proteobacteria, Cyanobacteria, Firmicutes, Acidobacteria, and Deinococcus-Thermus. These five dominant bacteria accounted for more than 80% of the total bacterial communities in all samples, but their relative abundance varied in the process of sweating. The most abundant phyla were Proteobacteria (23.17–99.05%), Cyanobacteria (0.05–56.39%), Firmicutes (0.61–50.69%), Actinobacteria (0–7.43%), and Deinococcus-Thermus (0.0–4.41%) (Fig. 2c). The fungal phyla with high relative abundance were Ascomycota and Basidiomycota. During the entire sweating process, the Ascomycete first increased and then decreased in the early stage (0 day–3 dp) and occupied an absolute advantage in the late stage of sweating (4 da–6 day), at which time the abundance was over 99% (Fig. 2d). Meanwhile, we analyzed the relative abundance at the class, order, family and genus levels (Additional file 1: Figures S1 and S2). Among them, the predominant bacterial genera were *Klebsiella*, *Enterobacter*, *Enterococcus*, *Actinomycetospora*, *Bacillus*, and the predominant fungus genera were *Capnobotryella*, *Devriesia*, *Candida*, *Aspergillus* and *Devriesia*.

### Analysis of differential microbial communities

**Fig. 3:**
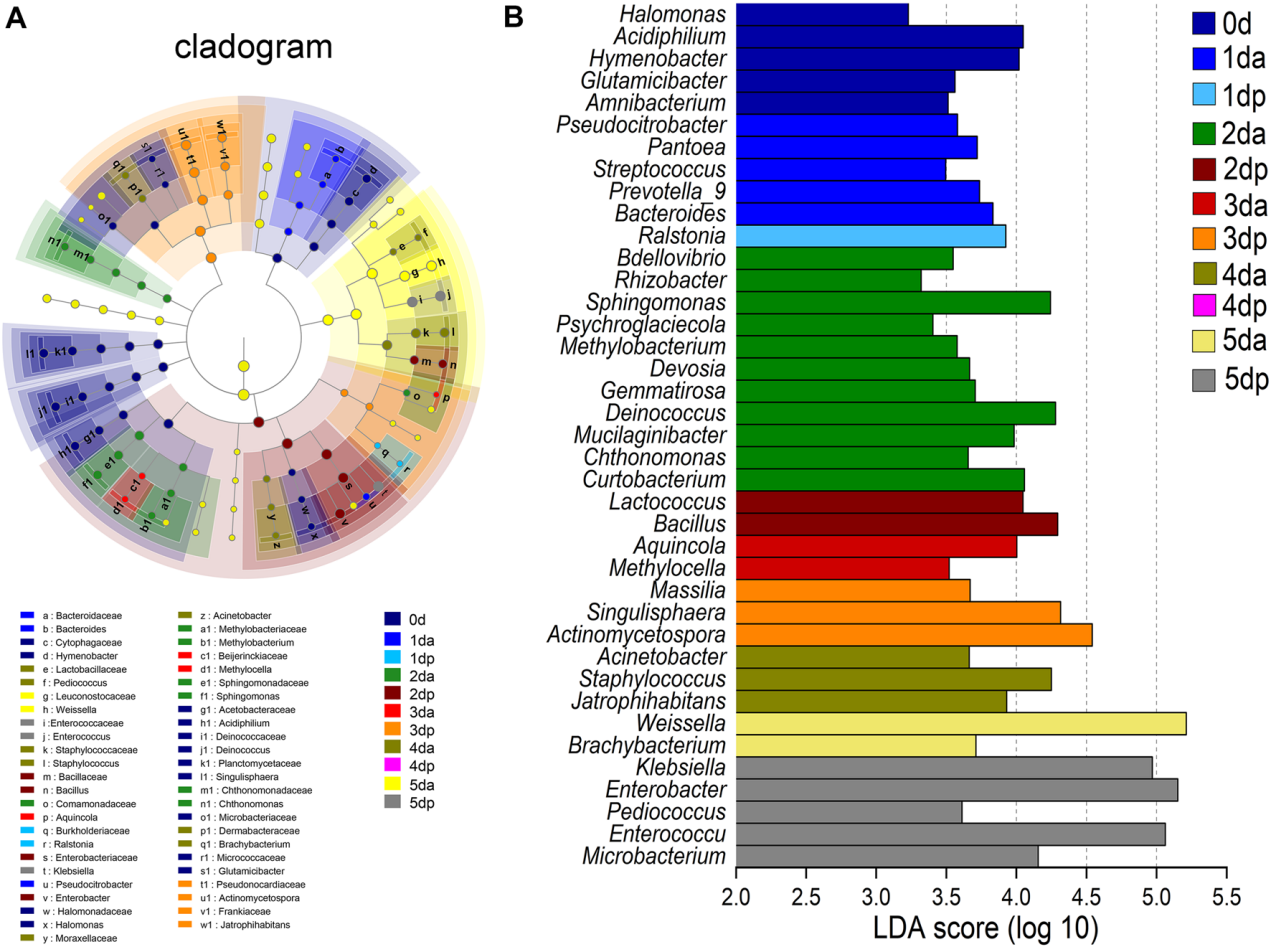
Linear discriminant effect size -identified differentially abundant taxa at different time points during the “sweating” phase of *M. officinalis*. **A** Enriched taxa reaching a linear discriminant analysis (LDA) significance threshold of 3.0 or greater in bacterial communities at different time points during the process of “sweating” of *M. officinalis*. **B** Cladograms are shown for the LDA values exceeding 3.0 for clarity. The relative abundance of taxa is more than 0.05% in each sample. Small circles and shading with different colors in the diagram represent the abundance of taxa in the different timepoints samples. Yellow circles present nonsignificant differences in abundance between the samples of a particular taxon. Each circle diameter is proportional to the taxon’s abundance

**Fig. 4:**
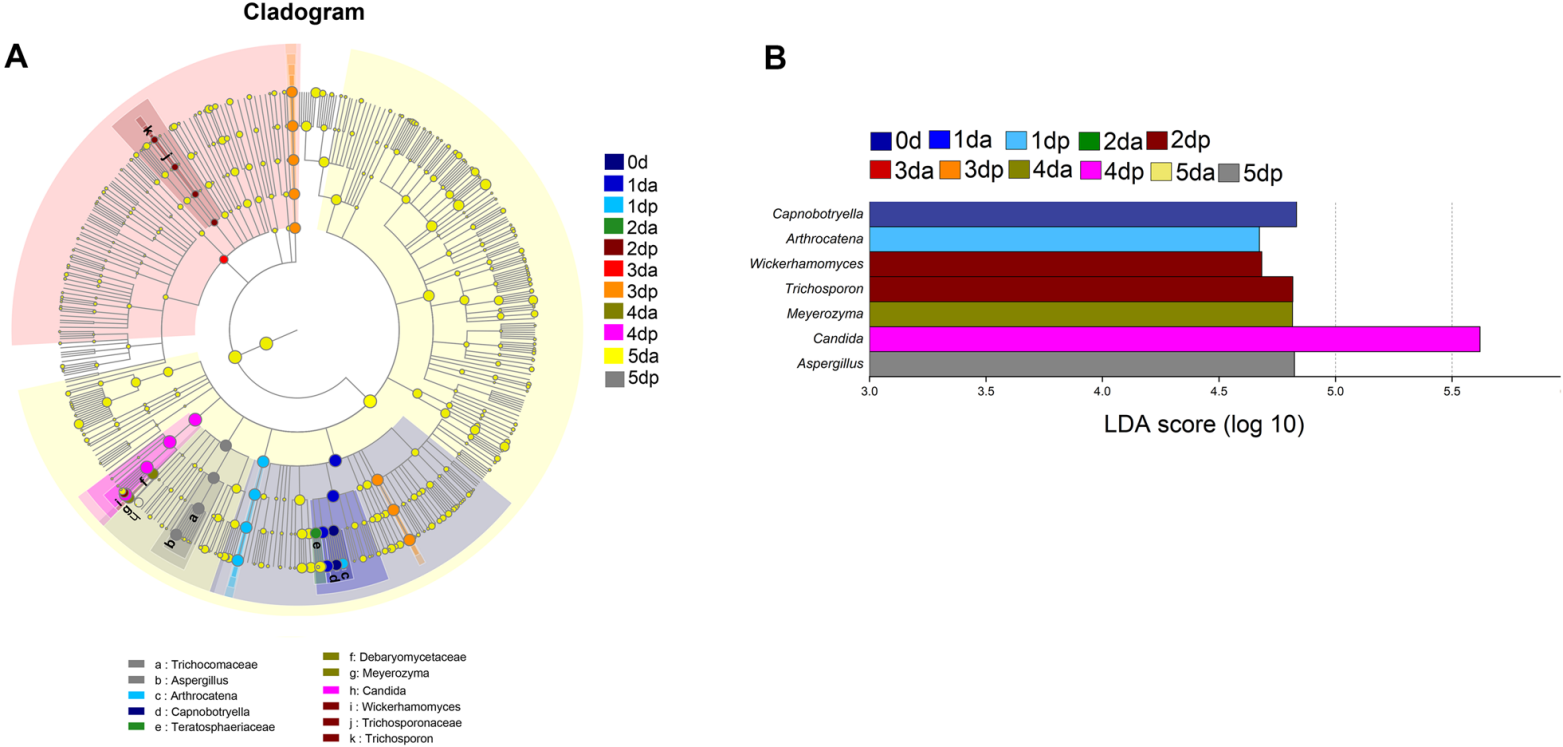
Linear discriminant effect size -identified differentially abundant taxa at different time points during the “sweating” phase of *M. officinalis*. **A** Enriched taxa reaching a linear discriminant analysis (LDA) significance threshold of 3.0 or greater in funal communities at different time points during the process of “sweating” of *M. officinalis*. **B** Cladograms are shown for the LDA values exceeding 3.0 for clarity. The relative abundance of taxa is more than 0.05% in each sample. Small circles and shading with different colors in the diagram represent the abundance of taxa in the different timepoints samples. Yellow circles present nonsignificant differences in abundance between the samples of a particular taxon. Each circle diameter is proportional to the taxon’s abundance

In addition to identifying specialized communities in samples, we used the LEfSe tool on the I-Sanger platform, which analyzes microbial community data at any clade. Due to the complexity and large number of OTUs that were detected in this study, the cladograms for the taxa with a relative abundance exceeding 0.05% in each sample were considered, and statistical analysis was carried out from the phylum to the genus level (Figs. 3A and 4A). To determine the microbial taxa with a significant difference in the abundance of Houpo with different sweating timepoints, we performed biomarker analysis using the linear discriminant analysis (LDA) effect size (LEfSe) method. The enriched taxa with an LDA significance threshold of 3.0 are shown in Figs. 3B and 4B.

As shown in LEfSe Bar, 5, 5, 1, 11, 2, 2, 3, 3, 2 and 5 bacterial groups were enriched as biomarkers in the 0 d, 1 da, 1 dp, 2 da, 2 dp, 3 da, 3 dp, 4 da, 5 da, 5 dp and 6 day samples, respectively, from the phylum to the genus level. For bacteria, the following phyla and genera were enriched in 0 day: Proteobacteria, Bacteroidetes, Actinobacteria, *Halomonas*, *Acidiphilium*, *Hymenobacter*, *Glutamicibacter* and *Amnibacterium*. The enriched taxa at 1 da mainly belonged to Proteobacteria, Firmicutes, Bacteroidetes, *Pseudocitrobacter*, *Pantoea*, *Streptococcus*, *Prevotella*_9 and *Bacteroides*, and *Ralstonia* was enriched in the 1 dp sample. In the 2 da sample, enriched bacterial lineages, such as *Bdellovibrio*, *Rhizobacter*, *Sphingomonas*, *Psychroglaciecola*, *Methylobacterium* mainly belonged to Proteobacteria. In the 2 dp and 3 da samples, Firmicutes and Proteobacteria were mainly enriched, respectively. *Singulisphaera* and *Actinomycetospora* were enriched in the 3 dp sample. In the 4 da sample, *Jatrophihabitans* and *Staphylococcus* were mainly enriched, but no significant enrichment in bacteria was detected in the 4 dp sample. In the 5 da sample, the significantly enriched microbes belonged to 2 genera (*Weissella*, *Brachybacterium*). At the end of sweating, 5 bacteria (*Klebsiella*, *Enterobacter*, *Pediococcus*, *Enterococcus and Microbacterium*) were detected as significantly enriched in the 5 dp sample. For fungi, 1, 1, 1, 2, 1 and 1 fungal groups were enriched as biomarkers in the 0 day, 1 dp, 2 dp, 4 da, 4 dp and 5 dp samples, respectively, from the phylum to the genus level, while no significant enrichment of fungi was detected at the genus clade in the 1 da, 3 da, 3 dp, 5 da samples. The following phyla were mainly enriched in Ascomycota, and the enrichment genera were *Capnobotryella*, *Arthrocatena*, *Wickerhamomyces*, *Candida* and *Aspergillus*. These results implied that the distinct compositions of the bacterial and fungal community changed in the process of Houpo sweating.

### Relationship between microbial community and content of four compounds

**Fig. 5:**
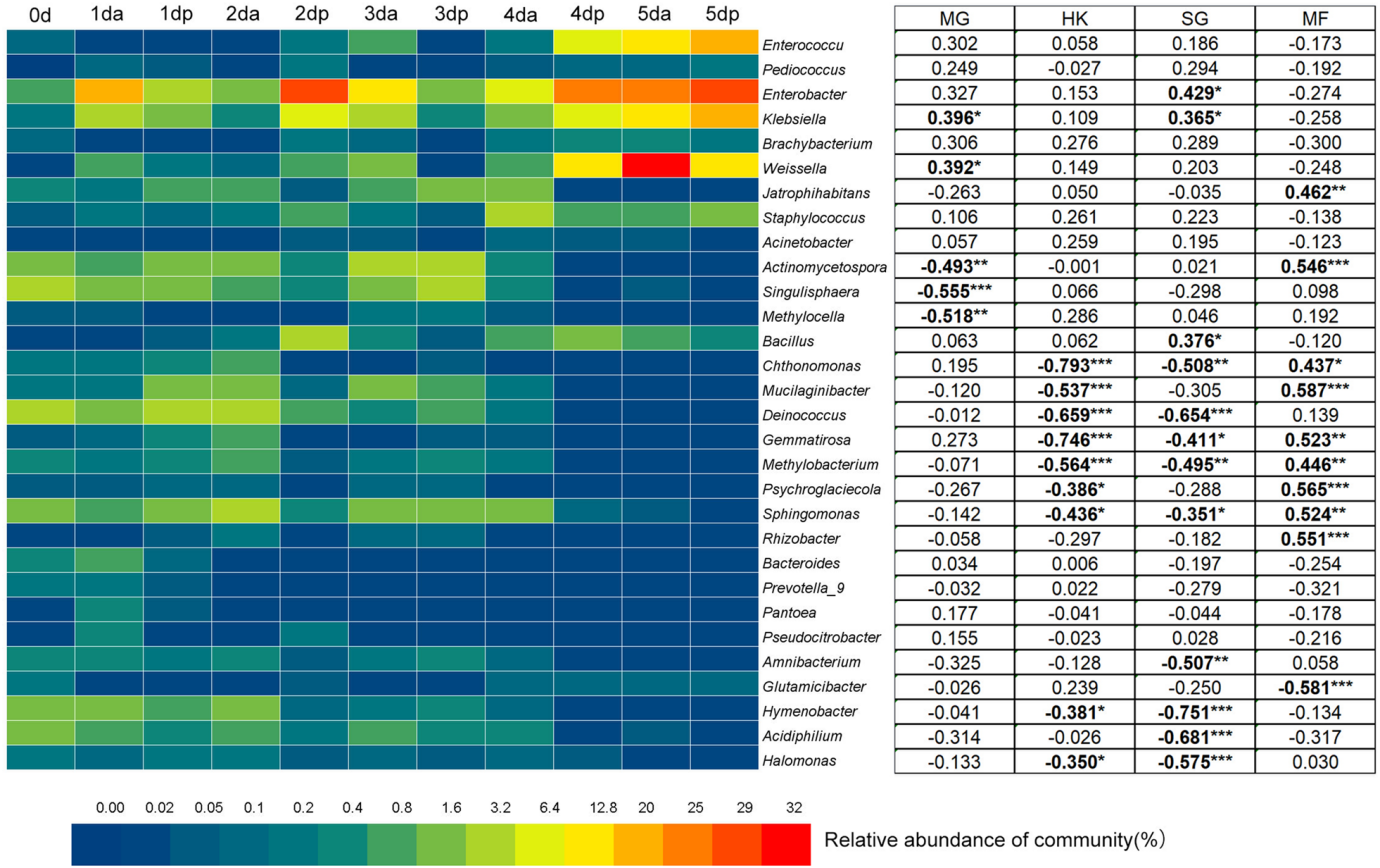
The relative abundance of bacterial communities > 0.05% in each sample at the genus level detected by LEfSe as biomarker and their Pearson’s correlation coefficients with total phenols content. Data are mean values of n = 3; significant correlation coefficients are noted in bold font where *P *< 0.05. *, ** and *** denote significant differences at *P *< 0.05, *P* < 0.01 and *P *< 0.001, respectively

**Fig. 6:**
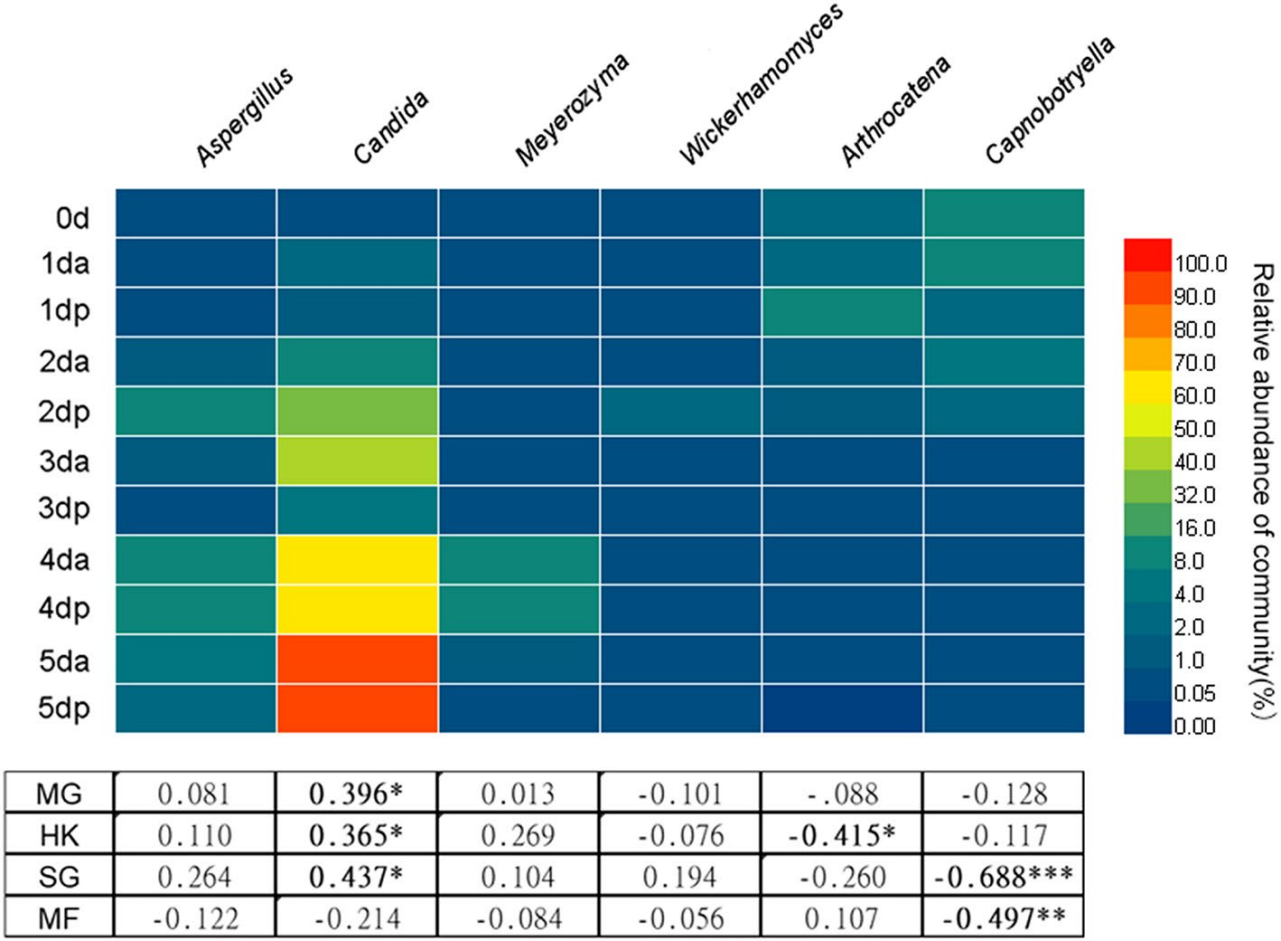
The relative abundance of fungal communities > 0.05% in each sample at the genus level detected by LEfSe as biomarker and their Pearson’s correlation coefficients with total phenols content. Data are mean values of n = 3; significant correlation coefficients are noted in bold font where *P *< 0.05. *, ** and *** denote significant differences at *P* < 0.05, *P* < 0.01 and *P *< 0.001, respectively

Sweating changed the microbial community structures and content of four main compounds. The heatmap revealed that the proportional distribution of the enriched dominant bacteria and fungi, as indicated by the high number of biomarkers in the process of sweating Houpo at the genus level, is shown in Figs. 5 and 6. The relative abundance of *Enterococcus*, *Enterobacter*, *Klebsiella*, *Weissella*, *Wickerhamomyces*, *Meyerozyma*, *Aspergillus*, and *Candida* significantly increased in the process of sweating (P < 0.05). In contrast, the relative abundances of *Actinomycetospora*, *Singulisphaera*, *Sphingomonas*, *Amnibacterium*, *Hymenobacter*, *Acidiphilium*, *Halomonas*, *Capnobotryella*, and *Arthrocatena* significantly declined in the process of sweating (P < 0.05).

Pearson’s correlation analysis revealed that the content of four compounds was related to the relative abundance (> 0.05%) of specific microbes (Figs. 5 and 6). In terms of bacteria, the content of MG was significantly positively correlated with the relative abundance of *Weissella* (P = 0.024), *Enterococcus* (P = 0.029), and *Klebsiella* (P = 0.022), while negatively correlated with *Actinomycetospora* (P = 0.004), *Singulisphaera* (P = 0.001), and *Methylocella* (P = 0.002). Meanwhile, the content of HK was significantly negatively correlated with the relative abundance of *Chthonomonas* (P = 0.000), *Mucilaginibacter* (P = 0.001), *Deinococcus* (P = 0.000), *Gemmatirosa* (P = 0.000), *Methylobacterium* (P = 0.001), *Psychroglaciecola* (P = 0.027), *Sphingomonas* (P = 0.011), *Hymenobacter* (P = 0.029), and *Halomonas* (P = 0.046). The SG content was significantly positively correlated with the relative abundance of *Enterobacter* (P = 0.013), *Klebsiella* (P = 0.037) and *Bacillus* (P = 0.031) but negatively correlated with the relative abundance of *Chthonomonas* (P = 0.003), *Deinococcus* (P = 0.000), *Gemmatirosa* (P = 0.018), *Methylobacterium* (P = 0.003), *Sphingomonas* (P = 0.045), *Amnibacterium* (P = 0.003), *Hymenobacter* (P = 0.000), *Acidiphilium* (P = 0.000) and *Halomonas* (P = 0.000). The MF content was significantly positively correlated with the relative abundance of *Jatrophihabitans* (P = 0.007), *Actinomycetospora* (P = 0.001), *Chthonomonas* (P = 0.011), *Mucilaginibacter* (P = 0.000), *Gemmatirosa* (P = 0.002), *Methylobacterium* (P = 0.009), *Psychroglaciecola* (P = 0.001), *Sphingomonas* (P = 0.002) and *Rhizobacter* (P = 0.001) but negatively correlated with the relative abundance of *Glutamicibacter* (P = 0.000).

For fungi, the contents of MG, HK and SG were significantly positively correlated with the relative abundance of *Candida* (P < 0.05). While the content of HK was negatively correlated with the relative abundance of *Arthrocatena* (P < 0.05), the contents of SG and MF were negatively correlated with the relative abundance of Capnobotryella (P < 0.01). This study reveals the close relationship between the content of the main active compounds and microbial community structure in the process of sweating.

### Statistical analysis of differential metabolites of unsweated Houpo before and after co-culture with different bacterial solutions

The volcano plot can be used to quickly check the difference in the expression level of metabolites in the two groups of samples, as well as the statistical significance of the difference.

**Fig. 7:**
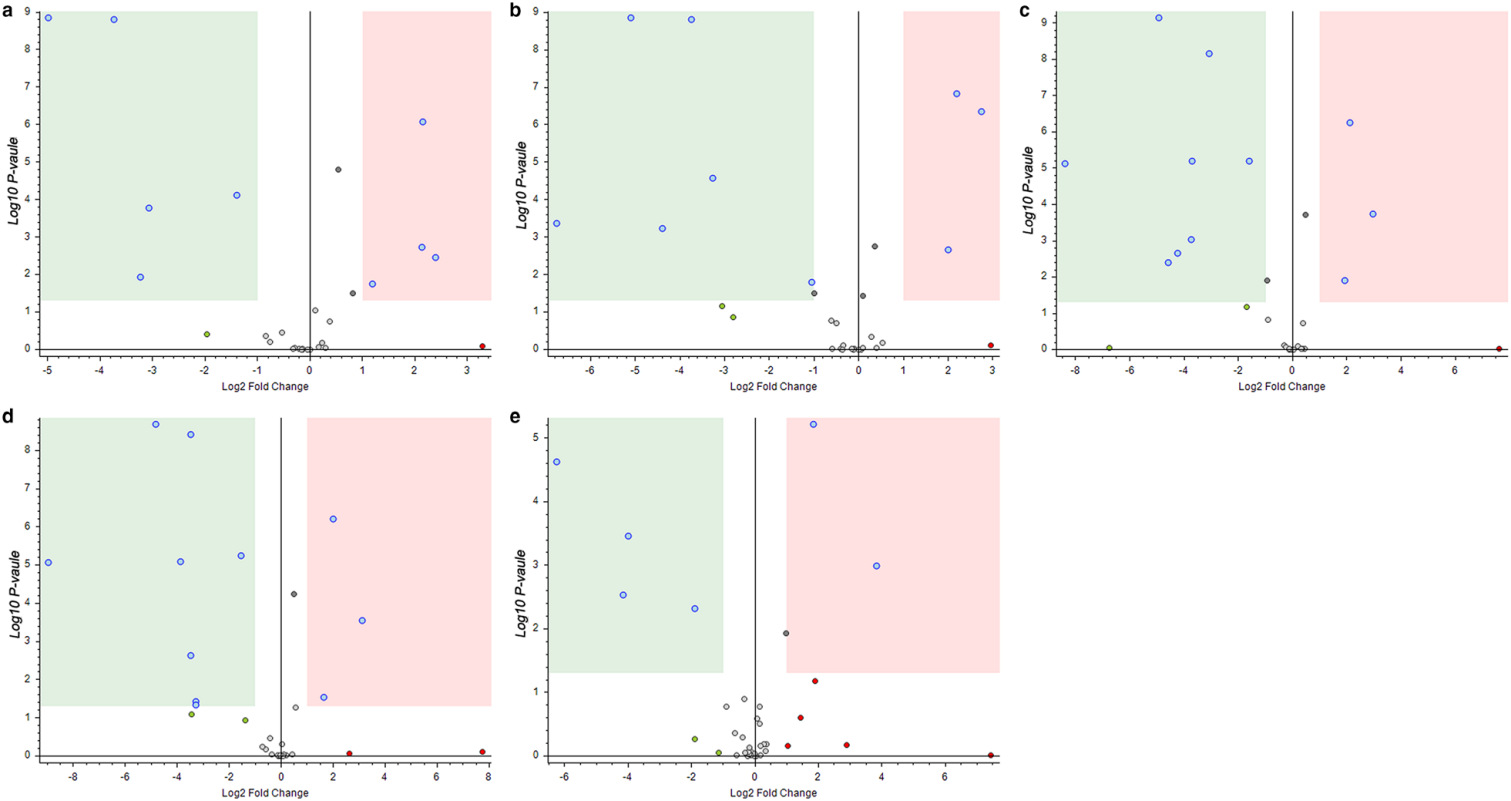
Volcano plots of differential metabolites of unsweated Houpo co-cultured with different bacterial solutions. The red area represents a significantly up-regulated and the green area represents significantly down-regulated, with P < 0.05, |Log2 FC| ≥ 1

After co-culture with different bacterial solutions, the metabolites of Houpo changed obviously (Fig. 7, Additional file 1: Tables S5 and S6). The volcano plots have shown that 4 metabolites of Houpo were significantly upregulated and 5 metabolites were significantly downregulated after treatment with E1 bacteria solution (Fig. 7a). After treatment with E2 bacterial solution, 3 metabolites were significantly upregulated and 6 metabolites were significantly downregulated (Fig. 7b). Meanwhile, 3 metabolites were significantly upregulated and 8 metabolites were significantly downregulated after K1 solution treatment (Fig. 7c), and 3 metabolites were significantly upregulated and 8 metabolites were significantly downregulated after treatment with K2 bacterial solution (Fig. 7d). After treatment with B bacteria solution, 2 metabolites were significantly upregulated and 4 metabolites were significantly downregulated (Fig. 7e).

## Discussion

In this study, we analyzed the dynamic variations of microbial communities and main active compounds (MG, HK, SG and MF) to determine the relationship of pivotal taxa with effective constituents in the process of sweating timepoints of *M. officinalis*. The contents of the main active compounds (MG, HK, SG and MF) increased to different degrees in the process of sweating, with the highest content at 4 dp. The diversity and abundance of bacterial and fungal communities vary greatly in the process of sweating, and samples from each timepoint had unique microbial populations. Additionally, the content of four main compounds (MG, HK, SG and MF) were closely related to the abundance of the microbial community. Finally, 5 different species of bacteria were selected to co-culture with unsweated Houpo, and their metabolites also changed significantly before and after the reaction. This study provided useful information on the microbial community and the content of four main compounds throughout the process of sweating, which contributed to harnessing the microbes to improve the quality of herbal medicines.

In the present study, we found that the total content of MG and HK increased by up to 3.75%, which was significantly higher than those of the unsweated samples (by 2.79%), with an increase of 32%. It has been reported that sweating can greatly increase the total amount of MG and HK by up to 40% [35]. Liu et al. [59] found that the content of MF decreased and SG increased after sweating, which was inconsistent with this study showing that the content of SG and MF increased. Meanwhile, this study detected the metabolites of different bacteria and unsweated Houpo before and after co-culture by UPLC-QE Orbitrap MS. It was found that the relative peak area of the four main active components did not change significantly, but there were some other metabolites upregulated and downregulated (Fig. 7, Additional file 1: Tables S6 and S7). This implies that in the process of Houpo sweating, there was a specific subset of species in the bacterial community contributes to the formation of the characteristics compounds and secondary metabolites [60]. The dynamic change of microbial community structure in the process of sweating may have a great influence on the quality of Houpo. Our results supported the first hypothesis that sweating will impact the quality of Houpo.

In this study, we found that the changes in microbial community structure had different contributions to the content of medicinal materials (Fig. 2a, b). The process of Houpo sweating can be understood as the participation of microorganisms in the transformation of TCM. Microbial transformation refers to the production of chemicals by microorganisms that react with or modify the active components of TCM and increase the content of active components [61]. In the process of sweating, most of the active components in plant tissues were also fully dissolved and transformed, which greatly increases the content of active components and thus enhances the efficacy of drugs [62]. In the early stage of sweating, the change in the internal temperature and water had a great influence on the microbial community, which was more beneficial to the growth and reproduction of microorganisms, and the microbial diversity and abundance were much higher than those in the later stage of sweating (Table 1). In the process of sweating, the diversity of bacteria and fungi at 2 da was significantly higher than those of other samples, and the dominant species were mainly unknown species (Additional file 1: Figures S1, S2). Meanwhile, LEfSe results showed that compared to the other timepoints, there were more bacterial species (Fig. 3) with significant differences at 2 da, but there was no significant difference in fungi (Fig. 4), and the content of MG and MF increased significantly, suggesting that the richer the microbial population, the more species that grow and propagate. The metabolites produced by different species of bacteria are generally different. The difference in the microecological structures of medicinal plants of the same host can lead to the diversity of microecological functions. Different microecological communities of TCM affect the metabolism and chemical composition of medicinal materials in various ways [63]. However, at 4 dp, there was no significant difference in bacteria, while the fungus with a significant difference was *Candida*, and its abundance increased significantly, as high as 96.56% (Additional file 1: Figures S1, S2). *Candida* belongs to the phylum Ascomycota, which were dominant in the process of sweating (Fig. 2c, d), and *Candida* was the most abundant endophytic fungi [64, 65]. Previous studies have also shown that the members of Ascomycota are composed of saprotrophic fungi, which are significantly affected by the degradation of plant species and straw residues [66, 67]. At 4 dp, the contents of MG, HK and SG were significantly higher than those of other samples, and the content of MF was relatively low. It is speculated that the contents of MG, HK and SG may be related to the high abundance of *Candida*.

In our study, the contents of MG, HK and SG were negatively correlated with the content of MF. Pearson’s correlation analysis revealed that *Enterobacter* (P < 0.05), *Klebsiella* (P < 0.05), *Weissella* (P < 0.05), *Bacillus* (P < 0.05) and *Candida* (P < 0.05) were significantly positively correlated with the content of MG and SG, which would be more conducive to improving the quality of Houpo (Figs. 5 and 6). With the development of sweating, *Klebsiella*, *Enterococcus* and *Enterobacter* gradually became the dominant species. In additional, *Klebsiella* was found during the fermentation of soy sauce and other fermented foods [68, 69]. Several studies have shown that *Klebsiella* species can ferment a variety of substrates, including pentoses, hexoses, and disaccharides, producing ethanol, butanediol and propanediol [70–72]. These substrates are readily used as carbon sources by various microorganisms during growth [73]. *Enterococcus* was also one of the dominant bacteria isolated from the traditional medicinal plant *Tridax procumbens* Linn. [74]. Previous studies has been reported that *Enterococcus* plays a significant role in flavor development and used as probiotics nowadays [75]. Meanwhile, *Aspergillus* was not only the dominant fungus in the whole sweating process, but also a kind of strain that has been used to transform TCM [76]. Pang et al. showed that *Aspergillus*, *Penicillium* and *Fusarium* were the dominant endophytic fungi in *M. officinalis*, and the dominant flora of endophytes were different from different sampling sites [77].

Thus, *Aspergillus*, *Klebsiella*, *Enterococcus*, *Bacillus* might group together to form a core microbiome that contributes to the production of certain key metabolites or change the content of certain metabolites in the process of Houpo sweating. The antibiotics, hormones, inducers and other active substances synthesized by these endophytic bacteria influence the synthesis of bioactive metabolites such as terpenoids, flavonoids, lignans by the host [78, 79]. It can degrade macromolecular substances by secreting glycosidase, glucose amylase, lactic acid enzyme and other extracellular enzymes [80]. The increase in MG and HK content after sweating may be related to the participation of microorganisms in the transformation of Houpo, thereby increasing the content of active components [81]. In addition, a negative correlation revealed that *Singulisphaera*, *Mucilaginibacter*, and *Psychroglaciecola* (Fig. 6) could be potential antagonists that reduce the quality of *M. officinalis*. It may be that some microbial transformation reduces the content of active components in TCM by decomposition or modification [82]. The formation process of secondary metabolites of medicinal plants is inhibited and regulated by the microecological flora of medicinal plants, and even the biosynthesis of some key steps is mainly completed by the microecological flora of plants [83]. It can be concluded that endophytic fungi can produce the same or similar chemical composition as the host to a certain extent, which can alleviate the shortage of plant resources [77].

## Conclusions

In summary, our study presented the bacterial and fungal community dynamics in relation to the quality of Houpo in the process of sweating and determined the pivotal microbial taxa related to the content of four main active compounds. The beneficial microorganisms and pathogenic bacteria screened in the study provided the basis for improving the quality of *M. officinalis*. In the long run, our work would be of great significance to understand the relationship between microorganisms and the quality of TCM and to improve the quality of TCM by using screened microorganisms.

## Supplementary information

**Additional file 1: Table S1.** Calibration curve of four tested compounds. **Table S2.** Stability, repeatability, precision and recovery rate of four tested compounds. **Table S3.** Quality control of bacterial. **Table S4.** Quality control of fungal. **Table S5.** Differential metabolites of unsweated Houpo before and after co-culture with different bacterial solutions. **Table S6.** Relative peak area of differential metabolites. **Table S7.** The relative peak area of four main active components in Houpo treated with different bacterial liquid. **Figure S1.** The relative abundance (> 0.10%) of bacteria taxa at four levels. (A)the relative abundance of bacteria at class level. (B)the relative abundance of bacteria at order level. (C) the relative abundance of bacteria at family level. (D) the relative abundance of bacteria at genus level. **Figure S2.** The relative abundance (> 0.10%) of fungus at four levels. (A) the relative abundance of fungus at class level. (B) the relative abundance of fungus at order level. (C) the relative abundance of fungus at family level. (D) the relative abundance of fungus at genus level.

## Data Availability

The 16S rRNA and 18S rRNA sequences used in this manuscript have been submitted to the NCBI and the Accession numbers are SRP: 534027 and 492971. Most of the data generated of analyzed during the study are included in this article and its Additional file 1.

## Abbreviations

*M. officinalis*: *Magnolia officinalis* Rehd. et Wils
TCMM: Traditional Chinese Medicinal Materials
da: day AM sample
dp: day PM sample
HPLC: high-performance liquid chromatography
MG: magnolol
HK: honokiol
MF: magnoflorine
SG: syringin
CP 2015: the Chinese Pharmacopoeia of version 2015
PCoA: principal coordinates analysis
OUTs: operational taxonomic units
LDA: linear discriminant analysis
