# Supplementary material for: Variation in the microbial community contributes to the improvement of the main active compounds of Magnolia officinalis Rehd. et Wils in the process of sweating
Source: Chin Med. 2019 Oct 22;14:45. doi: 10.1186/s13020-019-0267-4 (PMC6806532; doi:10.1186/s13020-019-0267-4)
Supplement: Supplementary file 1 — Additional file 1: Table S1. Calibration curve of four tested compounds. Table S2. Stability, repeatability, precision and recovery rate of four tested compounds. Table S3. Quality control of bacterial. Table S4. Quality control of fungal. Table S5. Differential metabolites of unsweated Houpo before and after co-culture with different bacterial solutions. Table S6. Relative peak area of differential metabolites. Table S7. The relative peak area of four main active components in Houpo treated with different bacterial liquid. Figure S1. The relative abundance (> 0.10%) of bacteria taxa at four levels. (A)the relative abundance of bacteria at class level. (B)the relative abundance of bacteria at order level. (C) the relative abundance of bacteria at family level. (D) the relative abundance of bacteria at genus level. Figure S2. The relative abundance (> 0.10%) of fungus at four levels. (A) the relative abundance of fungus at class level. (B) the relative abundance of fungus at order level. (C) the relative abundance of fungus at family level. (D) the relative abundance of fungus at genus level. [file 13020_2019_267_MOESM1_ESM.docx]

# Results

The linearity of all calibration curves were *R*^2^>0.999, and the established HPLC methods could be used for accurate and sensitive quantitative analysis (Table S1 and S2).

**Table S1** **Calibration curve of** **four tested compounds** (n=3)

| **Reference substance** | **Calibration curve** | **R^2^** | **Linear range(μg/mL)** |
| --- | --- | --- | --- |
| Honokiol | y=0.1125x+0.0059 | 0.9999 | 4.8-38.4 |
| Magnolol | y=0.1176x+0.0065 | 0.9999 | 8.0-64.0 |
| Syringin | y=0.3622x-0.5849 | 0.9997 | 1.0-80.0 |
| Magnoflorine | y=0.233x+0.2460 | 0.9994 | 2.5-8.0 |

**Table S2 Stability, repeatability, precision and recovery rate of four tested compounds**

| **Compound** | **Stablility RSD(%)(n=6)** | **Repeatability RSD(%)(n=6)** | **Precision RSD(%)(n=6)** | **Recovery RSD(%)(n=3)** |
| --- | --- | --- | --- | --- |
| Honokiol | 0.95 | 1.72 | 0.14 | 2.92 |
| Magnolol | 0.51 | 1.05 | 0.08 | 1.58 |
| Syringin | 3.47 | 2.07 | 0.21 | 3.12 |
| Magnoflorine | 0.92 | 0.95 | 0.38 | 4.57 |

**Table S3** **Quality control of bacterial (n=33)**

| **Sample** | **Raw reads** | **Average length** | **Total bases** | **Q30** | **Q20** | **Primer** |
| --- | --- | --- | --- | --- | --- | --- |
| 0 d-1 | 44201 | 429.5714803 | 18987489 | 94.07493534 | 98.19966189 | 338F_806R |
| 0 d-2 | 45707 | 429.9480605 | 19651636 | 93.89018299 | 98.12090454 | 338F_806R |
| 0 d-3 | 50170 | 430.4601754 | 21596187 | 94.02680668 | 98.15487799 | 338F_806R |
| 1 da-1 | 43669 | 435.0185486 | 18996825 | 93.50483041 | 98.04955828 | 338F_806R |
| 1 da-2 | 44444 | 436.3071281 | 19391234 | 93.76778703 | 98.09272066 | 338F_806R |
| 1 da-3 | 44423 | 435.815546 | 19360234 | 93.75226043 | 98.10494543 | 338F_806R |
| 1 dp-1 | 54435 | 431.0504822 | 23464233 | 94.07451759 | 98.1825743 | 338F_806R |
| 1 dp-2 | 40091 | 430.1737797 | 17246097 | 93.56686327 | 97.99462452 | 338F_806R |
| 1 dp-3 | 46462 | 431.1072274 | 20030104 | 94.39736808 | 98.29282963 | 338F_806R |
| 2 da-1 | 41974 | 431.1738219 | 18098090 | 93.80555075 | 98.11527625 | 338F_806R |
| 2 da-2 | 61570 | 431.6868605 | 26578960 | 93.85696054 | 98.09180645 | 338F_806R |
| 2 da-3 | 59049 | 430.5822622 | 25425452 | 93.48406471 | 97.98927468 | 338F_806R |
| 2 dp-1 | 45432 | 441.0190835 | 20036379 | 93.8983486 | 98.18472689 | 338F_806R |
| 2 dp-2 | 41781 | 437.9434911 | 18297717 | 93.61635662 | 98.04128023 | 338F_806R |
| 2 dp-3 | 40048 | 436.6132891 | 17485489 | 94.23085051 | 98.26759206 | 338F_806R |
| 3 da-1 | 55916 | 431.8220366 | 24145761 | 94.17685365 | 98.19061822 | 338F_806R |
| 3 da-2 | 36367 | 439.8602854 | 15996399 | 94.56504555 | 98.38522407 | 338F_806R |
| 3 da-3 | 45408 | 430.1159487 | 19530705 | 94.60116263 | 98.33282516 | 338F_806R |
| 3 dp-1 | 55263 | 430.84695 | 23809895 | 94.26467861 | 98.23528411 | 338F_806R |
| 3 dp-2 | 54354 | 429.8040439 | 23361569 | 94.59426291 | 98.33554416 | 338F_806R |
| 3 dp-3 | 46302 | 429.6865362 | 19895346 | 94.24502595 | 98.24282021 | 338F_806R |
| 4 da-1 | 43013 | 429.0951805 | 18456671 | 94.59203667 | 98.37146146 | 338F_806R |
| 4 da-2 | 46526 | 432.9472768 | 20143305 | 94.37734771 | 98.242071 | 338F_806R |
| 4 da-3 | 38436 | 434.8879436 | 16715353 | 94.66613119 | 98.40072178 | 338F_806R |
| 4 dp-1 | 36480 | 436.3648026 | 15918588 | 94.7704721 | 98.4324866 | 338F_806R |
| 4 dp-2 | 35731 | 445.2467325 | 15909111 | 94.40738706 | 98.33050382 | 338F_806R |
| 4 dp-3 | 38585 | 446.5390696 | 17229710 | 94.46954708 | 98.32992546 | 338F_806R |
| 5 da-1 | 36467 | 449.6155702 | 16396131 | 94.11654493 | 98.29445129 | 338F_806R |
| 5 da-2 | 35665 | 448.8203561 | 16007178 | 94.38002126 | 98.32110944 | 338F_806R |
| 5 da-3 | 39098 | 447.2698859 | 17487358 | 94.47892014 | 98.35331901 | 338F_806R |
| 5 dp-1 | 57659 | 449.9550634 | 25943959 | 94.61105377 | 98.39758843 | 338F_806R |
| 5 dp-2 | 40672 | 449.5527144 | 18284208 | 94.1621863 | 98.23391858 | 338F_806R |
| 5 dp-3 | 38391 | 449.5497382 | 17258664 | 94.66686993 | 98.4068987 | 338F_806R |

**Table S4 Quality control of fungal (n=33)**

| **Sample** | **Raw reads** | **Average length** | **Total bases** | **Q30** | **Q20** | **Primer** |
| --- | --- | --- | --- | --- | --- | --- |
| 0 d-1 | 73843 | 256.08977 | 18910437 | 97.912666 | 99.193747 | ITS1F_ITS2R |
| 0 d-2 | 65676 | 257.25802 | 16895678 | 97.936431 | 99.192758 | ITS1F_ITS2R |
| 0 d-3 | 70130 | 257.36859 | 18049259 | 97.962332 | 99.220782 | ITS1F_ITS2R |
| 1 da-1 | 70936 | 249.3619 | 17688736 | 98.299455 | 99.372205 | ITS1F_ITS2R |
| 1 da-2 | 65324 | 252.96706 | 16524820 | 98.358009 | 99.404018 | ITS1F_ITS2R |
| 1 da-3 | 71678 | 250.75792 | 17973826 | 98.37423 | 99.394464 | ITS1F_ITS2R |
| 1 dp-1 | 69646 | 249.67445 | 17388827 | 98.393905 | 99.424573 | ITS1F_ITS2R |
| 1 dp-2 | 64565 | 257.53927 | 16628023 | 98.450098 | 99.457813 | ITS1F_ITS2R |
| 1 dp-3 | 72859 | 250.86998 | 18278136 | 98.479249 | 99.446864 | ITS1F_ITS2R |
| 2 da-1 | 67158 | 247.65687 | 16632140 | 98.50166 | 99.43932 | ITS1F_ITS2R |
| 2 da-2 | 69993 | 256.69437 | 17966809 | 98.171083 | 99.282288 | ITS1F_ITS2R |
| 2 da-3 | 71868 | 249.27059 | 17914579 | 98.520641 | 99.451514 | ITS1F_ITS2R |
| 2 dp-1 | 71649 | 251.01553 | 17985012 | 98.261436 | 99.35206 | ITS1F_ITS2R |
| 2 dp-2 | 71225 | 247.99568 | 17663492 | 98.570668 | 99.461879 | ITS1F_ITS2R |
| 2 dp-3 | 59926 | 246.45393 | 14768998 | 98.574575 | 99.473255 | ITS1F_ITS2R |
| 3 da-1 | 71061 | 256.69924 | 18241305 | 98.590633 | 99.485881 | ITS1F_ITS2R |
| 3 da-2 | 67959 | 235.30645 | 15991191 | 99.466137 | 99.803404 | ITS1F_ITS2R |
| 3 da-3 | 73125 | 250.70462 | 18332775 | 98.840317 | 99.57816 | ITS1F_ITS2R |
| 3dp-1 | 73912 | 255.7112 | 18900126 | 98.577009 | 99.49085 | ITS1F_ITS2R |
| 3 dp-2 | 69015 | 254.49696 | 17564108 | 98.698072 | 99.525857 | ITS1F_ITS2R |
| 3 dp-3 | 74151 | 256.26292 | 19002152 | 98.743869 | 99.555392 | ITS1F_ITS2R |
| 4 da-1 | 71524 | 237.90255 | 17015742 | 99.326083 | 99.740846 | ITS1F_ITS2R |
| 4 da-2 | 69178 | 255.40871 | 17668664 | 98.845153 | 99.569328 | ITS1F_ITS2R |
| 4 da-3 | 59834 | 240.91659 | 14415003 | 99.09547 | 99.660708 | ITS1F_ITS2R |
| 4 dp-1 | 69188 | 235.28829 | 16279126 | 99.423937 | 99.785277 | ITS1F_ITS2R |
| 4 dp-2 | 71087 | 235.45821 | 16738018 | 99.409142 | 99.769644 | ITS1F_ITS2R |
| 4 dp-3 | 69590 | 235.44475 | 16384600 | 99.352966 | 99.75665 | ITS1F_ITS2R |
| 5 da-1 | 71981 | 236.94554 | 17055577 | 99.372446 | 99.763397 | ITS1F_ITS2R |
| 5 da-2 | 73002 | 235.11071 | 17163552 | 99.439452 | 99.797559 | ITS1F_ITS2R |
| 5 da-3 | 71390 | 235.40798 | 16805776 | 99.434504 | 99.786502 | ITS1F_ITS2R |
| 5 dp-1 | 71690 | 242.58064 | 17390606 | 98.978253 | 99.608024 | ITS1F_ITS2R |
| 5 dp-2 | 49501 | 238.07295 | 11784849 | 99.302019 | 99.734481 | ITS1F_ITS2R |
| 5 dp-3 | 71445 | 244.02843 | 17434611 | 99.030193 | 99.632312 | ITS1F_ITS2R |

**Table S5 Differential metabolites of unsweated Houpo before and after co-culture with different bacterial solutions**

| **Groups** | **Checked** | **Name** | **Formula** | **Molecular Weight** | **RT [min]** | **Area (Max.)** |
| --- | --- | --- | --- | --- | --- | --- |
| E1 VS KB | TRUE | 4-Indolecarbaldehyde | C9 H7 N O | 145.0529 | 16.028 | 41301811 |
|  | TRUE | Cyclo(phenylalanyl-prolyl) | C14 H16 N2 O2 | 244.12141 | 15.29 | 25944290 |
|  | TRUE | L-Phenylalanine | C9 H11 N O2 | 165.07902 | 9.005 | 10608709 |
|  | TRUE | D-(+)-cellobiose | C12 H22 O11 | 342.11635 | 4.786 | 7851785 |
|  | TRUE | unknown | C10 H16 N2 O2 | 196.12141 | 11.74 | 6016147 |
|  | TRUE | α-Lactose | C12 H22 O11 | 359.14286 | 4.736 | 5811032 |
|  | TRUE | unknown | C9 H7 N O | 145.05297 | 12.723 | 4454490 |
|  | TRUE | Succinic acid | C4 H6 O4 | 118.02584 | 6.886 | 1623045 |
|  | TRUE | unknown | C26 H34 O11 | 544.1928 | 12.998 | 896347 |
| E2 VS KB | TRUE | 4-Indolecarbaldehyde | C9 H7 N O | 145.0529 | 16.028 | 41301811 |
|  | TRUE | 4-Guanidinobutyric acid | C5 H11 N3 O2 | 145.08532 | 5.105 | 36901271 |
|  | TRUE | Cyclo(phenylalanyl-prolyl) | C14 H16 N2 O2 | 244.12141 | 15.29 | 25944290 |
|  | TRUE | Cyclo(leucylprolyl) | C11 H18 N2 O2 | 210.13705 | 15.011 | 14238096 |
|  | TRUE | D-(+)-cellobiose | C12 H22 O11 | 342.11635 | 4.786 | 7851785 |
|  | TRUE | DL-Arginine | C6 H14 N4 O2 | 174.11188 | 4.538 | 7315636 |
|  | TRUE | unknown | C10 H16 N2 O2 | 196.12141 | 11.74 | 6016147 |
|  | TRUE | α-Lactose | C12 H22 O11 | 359.14286 | 4.736 | 5811032 |
|  | TRUE | Proline | C5 H9 N O2 | 115.06378 | 4.89 | 3008537 |
| K1 VS KB | TRUE | 4-Indolecarbaldehyde | C9 H7 N O | 145.0529 | 16.028 | 41301811 |
|  | TRUE | Cyclo(phenylalanyl-prolyl) | C14 H16 N2 O2 | 244.12141 | 15.29 | 25944290 |
|  | TRUE | val-leu-pro-val-pro | C31 H53 N7 O8 | 651.39631 | 13.699 | 11826240 |
|  | TRUE | L-Phenylalanine | C9 H11 N O2 | 165.07902 | 9.005 | 10608709 |
|  | TRUE | D-(+)-cellobiose | C12 H22 O11 | 342.11635 | 4.786 | 7851785 |
| K1 VS KB | TRUE | unknown | C10 H16 N2 O2 | 196.12141 | 11.74 | 6016147 |
|  | TRUE | α-Lactose | C12 H22 O11 | 359.14286 | 4.736 | 5811032 |
|  | TRUE | Proline | C5 H9 N O2 | 115.06378 | 4.89 | 3008537 |
|  | TRUE | Tyrosine | C9 H11 N O3 | 181.07373 | 6.124 | 2153260 |
|  | TRUE | Succinic acid | C4 H6 O4 | 118.02584 | 6.886 | 1623045 |
|  | TRUE | unknown | C26 H34 O11 | 544.1928 | 12.998 | 896347 |
| K2 VS KB | TRUE | 4-Indolecarbaldehyde | C9 H7 N O | 145.0529 | 16.028 | 41301811 |
|  | TRUE | Cyclo(phenylalanyl-prolyl) | C14 H16 N2 O2 | 244.12141 | 15.29 | 25944290 |
|  | TRUE | val-leu-pro-val-pro | C31 H53 N7 O8 | 651.39631 | 13.699 | 11826240 |
|  | TRUE | L-Phenylalanine | C9 H11 N O2 | 165.07902 | 9.005 | 10608709 |
|  | TRUE | D-(+)-cellobiose | C12 H22 O11 | 342.11635 | 4.786 | 7851785 |
|  | TRUE | unknown | C10 H16 N2 O2 | 196.12141 | 11.74 | 6016147 |
|  | TRUE | α-Lactose | C12 H22 O11 | 359.14286 | 4.736 | 5811032 |
|  | TRUE | Proline | C5 H9 N O2 | 115.06378 | 4.89 | 3008537 |
|  | TRUE | Succinic acid | C4 H6 O4 | 118.02584 | 6.886 | 1623045 |
|  | TRUE | Guanosine | C10 H13 N5 O5 | 283.09164 | 6.5 | 1261014 |
|  | TRUE | unknown | C26 H34 O11 | 544.1928 | 12.998 | 896347 |
| B VS KB | TRUE | Adenosine | C10 H13 N5 O4 | 267.09682 | 7.488 | 39052197 |
|  | TRUE | DL-Norleucine | C6 H13 N O2 | 131.09484 | 7.281 | 12617037 |
|  | TRUE | DL-Arginine | C6 H14 N4 O2 | 174.11188 | 4.538 | 7315636 |
|  | TRUE | unknown | C10 H16 N2 O2 | 196.12141 | 11.74 | 6016147 |
|  | TRUE | unknown | C6 H6 N2 O | 122.04837 | 7.614 | 2696706 |
|  | TRUE | Xanthine | C5 H4 N4 O2 | 152.03307 | 6.246 | 2314655 |

**Table S6 Relative peak area of differential metabolites (n=3)**

| **Name** | **Tyrosine** | **4-Guanidin**  **obutyric acid** | **unknown** | **Adenosine** | **DL-Arginine** | **Guanosine** | **unknown** | **Proline** | **Succinic acid** | **val-leu-pro--val-pro** |
| --- | --- | --- | --- | --- | --- | --- | --- | --- | --- | --- |
| Area: E1-1.raw (F4) | 0.00E+00 | 8.26E+07 | 2.42E+08 | 1.13E+07 | 6.86E+05 | 4.21E+05 | 7.00E+06 | 6.12E+07 | 2.92E+04 | 4.02E+05 |
| Area: E1-2.raw (F5) | 1.96E+07 | 4.28E+08 | 8.12E+07 | 1.19E+07 | 4.00E+05 | 2.75E+06 | 7.40E+06 | 1.88E+07 | 2.55E+04 | 4.38E+05 |
| Area: E1-3.raw (F6) | 2.76E+07 | 5.53E+08 | 1.00E+08 | 1.37E+07 | 3.91E+05 | 2.01E+06 | 8.60E+06 | 6.58E+06 | 1.95E+04 | 6.89E+05 |
| Area: E2-1.raw (F7) | 3.08E+07 | 1.19E+09 | 7.73E+07 | 1.25E+07 | 3.73E+05 | 8.96E+05 | 5.89E+06 | 9.34E+06 | 2.56E+04 | 4.60E+05 |
| Area: E2-2.raw (F8) | 2.80E+07 | 2.03E+09 | 6.95E+07 | 1.08E+07 | 1.90E+06 | 6.71E+05 | 5.22E+06 | 6.65E+06 | 2.67E+04 | 2.26E+05 |
| Area: E2-3.raw (F9) | 2.62E+07 | 1.94E+09 | 5.88E+07 | 1.01E+07 | 3.15E+06 | 3.90E+05 | 9.22E+06 | 4.39E+06 | 3.22E+04 | 2.81E+05 |
| Area: K1-1.raw (F13) | 2.60E+07 | 2.04E+08 | 7.88E+07 | 1.37E+07 | 3.45E+05 | 1.94E+06 | 4.15E+06 | 1.36E+07 | 2.21E+04 | 8.07E+05 |
| Area: K1-2.raw (F14) | 1.10E+07 | 1.47E+08 | 5.54E+08 | 1.59E+07 | 2.10E+06 | 6.05E+06 | 6.31E+06 | 1.10E+07 | 2.75E+04 | 2.94E+06 |
| Area: K1-3.raw (F15) | 8.61E+06 | 1.11E+08 | 6.74E+07 | 1.26E+07 | 8.10E+05 | 3.53E+06 | 7.43E+06 | 7.91E+06 | 2.49E+04 | 4.14E+06 |
| Area: K2-1.raw (F16) | 2.05E+07 | 1.50E+08 | 5.40E+07 | 1.59E+07 | 2.25E+06 | 6.34E+06 | 4.93E+06 | 1.71E+07 | 2.69E+04 | 1.74E+06 |
| Area: K2-2.raw (F17) | 3.25E+07 | 1.60E+08 | 8.90E+07 | 1.28E+07 | 9.00E+05 | 2.99E+06 | 3.61E+06 | 1.17E+07 | 2.13E+04 | 1.11E+06 |
| Area: K2-3.raw (F18) | 1.59E+07 | 1.13E+08 | 6.09E+07 | 1.43E+07 | 4.35E+05 | 3.60E+06 | 2.33E+06 | 7.12E+06 | 2.99E+04 | 1.79E+06 |
| Area: B-1.raw (F22) | 7.94E+07 | 3.25E+08 | 1.04E+08 | 6.22E+07 | 7.49E+06 | 1.57E+06 | 7.92E+06 | 5.45E+07 | 3.74E+04 | 2.84E+05 |
| Area: B-2.raw (F23) | 3.51E+07 | 3.67E+08 | 6.90E+07 | 5.65E+07 | 1.91E+07 | 3.30E+06 | 7.88E+06 | 4.87E+07 | 2.53E+04 | 3.25E+05 |
| Area: B-3.raw (F24) | 5.70E+07 | 3.86E+08 | 6.12E+07 | 3.66E+07 | 3.30E+07 | 4.40E+06 | 8.52E+06 | 1.71E+08 | 3.01E+04 | 1.12E+05 |
| Area: KB-1.raw (F25) | 6.53E+07 | 2.88E+08 | 5.72E+07 | 1.88E+09 | 2.65E+08 | 2.16E+07 | 1.36E+07 | 1.25E+08 | 2.63E+04 | 1.94E+08 |
| Area: KB-2.raw (F26) | 5.90E+07 | 2.87E+08 | 5.22E+07 | 1.78E+09 | 2.69E+08 | 2.19E+07 | 3.37E+06 | 1.25E+08 | 3.08E+04 | 1.47E+08 |
| Area: KB-3.raw (F27) | 6.87E+07 | 2.88E+08 | 5.40E+07 | 1.90E+09 | 3.05E+08 | 2.37E+07 | 9.54E+06 | 1.29E+08 | 2.86E+04 | 1.66E+08 |
| **Name** | **phenylalanyl-prolyl** | **Xanthine** | **α-Lactose** | **4-Indolecar-**  **baldehyde** | **leucylprolyl** | **DL-Norleucine** | **unknown** | **L-Pheny**  **lalanine** | **unknown** | **D-(+)-cellobiose** |
| Area: E1-1.raw (F4) | 1.40E+07 | 3.43E+07 | 4.73E+06 | 2.37E+06 | 6.21E+06 | 4.89E+06 | 2.54E+05 | 1.52E+05 | 2.84E+06 | 2.40E+05 |
| Area: E1-2.raw (F5) | 2.02E+07 | 5.91E+06 | 4.69E+06 | 2.27E+06 | 8.53E+06 | 6.89E+06 | 2.93E+05 | 1.09E+06 | 4.64E+06 | 2.22E+05 |
| Area: E1-3.raw (F6) | 2.59E+07 | 7.13E+07 | 4.42E+06 | 4.65E+06 | 1.19E+07 | 9.32E+06 | 3.63E+05 | 1.65E+06 | 5.86E+06 | 2.21E+05 |
| Area: E2-1.raw (F7) | 2.44E+07 | 4.23E+06 | 5.82E+06 | 1.71E+06 | 8.25E+06 | 9.44E+06 | 5.87E+05 | 1.75E+06 | 6.00E+06 | 2.74E+05 |
| Area: E2-2.raw (F8) | 1.84E+07 | 4.13E+06 | 4.38E+06 | 2.66E+06 | 5.93E+06 | 8.89E+06 | 5.44E+05 | 1.47E+06 | 4.77E+06 | 2.07E+05 |
| Area: E2-3.raw (F9) | 1.53E+07 | 4.14E+06 | 4.41E+06 | 2.07E+06 | 5.10E+06 | 8.83E+06 | 4.99E+05 | 1.38E+06 | 4.73E+06 | 2.07E+05 |
| Area: K1-1.raw (F13) | 1.74E+07 | 7.86E+06 | 4.49E+06 | 2.00E+06 | 9.90E+06 | 6.51E+06 | 2.64E+05 | 1.31E+06 | 4.88E+06 | 2.35E+05 |
| Area: K1-2.raw (F14) | 1.27E+07 | 7.76E+06 | 5.74E+06 | 1.53E+06 | 8.42E+06 | 3.32E+06 | 2.55E+05 | 5.48E+05 | 3.93E+06 | 1.20E+05 |
| Area: K1-3.raw (F15) | 2.22E+07 | 9.10E+06 | 5.61E+06 | 1.34E+06 | 1.12E+07 | 3.68E+06 | 2.12E+05 | 7.14E+04 | 4.58E+06 | 2.64E+05 |
| Area: K2-1.raw (F16) | 1.44E+07 | 8.07E+06 | 4.97E+06 | 1.28E+06 | 6.39E+06 | 2.84E+06 | 1.92E+05 | 1.06E+06 | 3.70E+06 | 2.24E+05 |
| Area: K2-2.raw (F17) | 7.73E+06 | 1.05E+07 | 5.35E+06 | 2.75E+06 | 1.06E+07 | 8.30E+06 | 2.66E+05 | 1.76E+06 | 5.47E+06 | 2.74E+05 |
| Area: K2-3.raw (F18) | 1.91E+07 | 8.61E+06 | 5.04E+06 | 1.38E+06 | 9.26E+06 | 4.50E+06 | 2.71E+05 | 5.79E+05 | 4.22E+06 | 2.48E+05 |
| Area: B-1.raw (F22) | 4.34E+06 | 5.18E+07 | 3.23E+07 | 2.69E+07 | 6.75E+06 | 1.33E+06 | 3.63E+05 | 9.20E+06 | 3.80E+06 | 6.48E+06 |
| Area: B-2.raw (F23) | 1.35E+07 | 4.85E+07 | 5.23E+07 | 4.13E+07 | 1.15E+07 | 3.16E+06 | 6.39E+05 | 4.50E+06 | 3.79E+06 | 7.52E+06 |
| Area: B-3.raw (F24) | 1.25E+07 | 5.32E+07 | 5.11E+07 | 4.07E+07 | 9.46E+06 | 5.31E+06 | 6.10E+05 | 6.85E+06 | 3.02E+06 | 2.81E+06 |
| Area: KB-1.raw (F25) | 3.51E+06 | 6.00E+06 | 2.59E+07 | 1.98E+07 | 1.42E+07 | 1.17E+07 | 8.96E+05 | 1.02E+07 | 1.10E+06 | 7.02E+06 |
| Area: KB-2.raw (F26) | 4.59E+06 | 6.27E+06 | 2.79E+07 | 1.78E+07 | 1.22E+07 | 1.12E+07 | 7.15E+05 | 9.35E+06 | 1.02E+06 | 7.85E+06 |
| Area: KB-3.raw (F27) | 5.26E+06 | 6.87E+06 | 2.92E+07 | 2.71E+07 | 1.21E+07 | 1.26E+07 | 7.62E+05 | 1.06E+07 | 1.04E+06 | 6.11E+06 |

**Table S7 The relative peak area of four main active components in Houpo treated with different bacterial liquid (n=3)**

| **Group** | **Honokiol Area** | **Magnolol Area** | **Magnoflorine Area** | **Syringin Area** |
| --- | --- | --- | --- | --- |
| KB-1 | 1.04E+11 | 2.20E+11 | 1.42E+10 | 6.53E+08 |
| KB-2 | 9.55E+10 | 2.10E+11 | 1.27E+10 | 6.30E+08 |
| KB-3 | 9.02E+10 | 2.00E+11 | 1.37E+10 | 7.21E+08 |
| E1-1 | 9.50E+10 | 1.95E+11 | 1.70E+10 | 2.76E+07 |
| E1-2 | 9.13E+10 | 1.92E+11 | 1.34E+10 | 1.17E+08 |
| E1-3 | 1.06E+11 | 2.21E+11 | 1.97E+10 | 7.19E+07 |
| E2-1 | 1.07E+11 | 2.09E+11 | 1.58E+10 | 6.29E+08 |
| E2-2 | 8.66E+10 | 1.84E+11 | 1.70E+10 | 5.79E+08 |
| E2-3 | 8.10E+10 | 1.67E+11 | 1.40E+10 | 6.05E+08 |
| K1-1 | 1.03E+11 | 2.16E+11 | 1.81E+10 | 3.25E+07 |
| K1-2 | 8.90E+10 | 1.93E+11 | 1.45E+10 | 4.93E+07 |
| K1-3 | 9.85E+10 | 2.16E+11 | 1.72E+10 | 3.90E+07 |
| K2-1 | 7.88E+10 | 1.74E+11 | 1.49E+10 | 5.94E+07 |
| K2-2 | 1.04E+11 | 2.18E+11 | 1.91E+10 | 3.54E+07 |
| K2-3 | 9.33E+10 | 2.06E+11 | 1.95E+10 | 7.02E+07 |
| B-1 | 7.52E+10 | 1.72E+11 | 1.47E+10 | 2.61E+08 |
| B-2 | 9.52E+10 | 2.06E+11 | 1.53E+10 | 7.32E+08 |
| B-3 | 8.60E+10 | 1.90E+11 | 1.62E+10 | 7.70E+08 |

**Figure S1**

**

**

**Figure S1** The relative abundance (>0.10%) of bacteria taxa at four levels. (A) relative abundance of bacteria at class level. (B) relative abundance of bacteria at order level. (C) relative abundance of bacteria at family level. (D) relative abundance of bacteria at genus level.

**Figure S2**





**Figure S2** The relative abundance (>0.10%) of fungus at four levels. (A) relative abundance of fungus at class level. (B) relative abundance of fungus at order level. (C) relative abundance of fungus at family level. (D) relative abundance of fungus at genus level.
